# Skin Exposure to Isocyanates: Reasons for Concern

**DOI:** 10.1289/ehp.9557

**Published:** 2006-11-28

**Authors:** Dhimiter Bello, Christina A. Herrick, Thomas J. Smith, Susan R. Woskie, Robert P. Streicher, Mark R. Cullen, Youcheng Liu, Carrie A. Redlich

**Affiliations:** 1 Exposure, Epidemiology and Risk Program, Harvard School of Public Health, Boston, Massachusetts, USA; 2 Department of Work Environment, University of Massachusetts Lowell, Lowell, Massachusetts, USA; 3 Department of Dermatology, Yale University School of Medicine, New Haven, Connecticut, USA; 4 Division of Applied Research and Technology, National Institute for Occupational Safety and Health, Cincinnati, Ohio, USA; 5 Occupational and Environmental Medicine Program, Yale University School of Medicine, New Haven, Connecticut, USA

**Keywords:** asthma, dermal exposure, isocyanates, sensitization, skin

## Abstract

**Objective:**

Isocyanates (di- and poly-), important chemicals used worldwide to produce polyurethane products, are a leading cause of occupational asthma. Respiratory exposures have been reduced through improved hygiene controls and the use of less-volatile isocyanates. Yet isocyanate asthma continues to occur, not uncommonly in settings with minimal inhalation exposure but opportunity for skin exposure. In this review we evaluate the potential role of skin exposure in the development of isocyanate asthma.

**Data sources:**

We reviewed the published animal and human literature on isocyanate skin-exposure methods, workplace skin exposure, skin absorption, and the role of skin exposure in isocyanate sensitization and asthma.

**Data extraction:**

We selected relevant articles from computerized searches on Medline, U.S. Environmental Protection Agency, Occupational Safety and Health Administration, National Institute for Occupational Safety and Health, and Google databases using the keywords “isocyanate,” “asthma,” “skin,” “sensitization,” and other synonymous terms, and our own extensive collection of isocyanate publications.

**Data synthesis:**

Isocyanate production and use continues to increase as the polyurethane industry expands. There is substantial opportunity for isocyanate skin exposure in many work settings, but such exposure is challenging to quantify and continues to be underappreciated. Isocyanate skin exposure can occur at work, even with the use of personal protective equipment, and may also occur with consumer use of certain isocyanate products. In animals, isocyanate skin exposure is an efficient route to induce sensitization, with subsequent inhalation challenge resulting in asthma-like responses. Several lines of evidence support a similar role for human isocyanate skin exposure, namely, that such exposure occurs and can contribute to the development of isocyanate asthma in certain settings, presumably by inducing systemic sensitization.

**Conclusions:**

Integrated animal and human research is needed to better understand the role of skin exposure in human isocyanate asthma and to improve diagnosis and prevention. In spite of substantial research needs, sufficient evidence already exists to justify greater emphasis on the potential risks of isocyanate skin exposure and the importance of preventing such exposures at work and during consumer use of certain isocyanate products.

Isocyanates, a group of reactive chemicals [with the functional group N = C = O (NCO)] used extensively in the production of numerous polyurethane foams, coatings, and a wide array of consumer products, are a major cause of occupational asthma worldwide. The polyurethane industry has expanded dramatically, along with the number of workers and consumers at risk for exposure. Inhalation has long been considered the primary route of isocyanate exposure, induction of sensitization, and asthma; research, practice, and regulation have focused almost exclusively on understanding and preventing inhalation exposures. Airborne isocyanate exposures have been reduced through improved controls and use of less-volatile isocyanates. Yet isocyanate asthma continues to occur, not uncommonly in work settings where measured isocyanate respiratory exposures are very low or nondetectable, but where there is opportunity for skin exposure.

It has been > 25 years since Karol et al. (1981) demonstrated in guinea pigs that skin contact with isocyanates could lead to sensitization and subsequent asthmatic responses following inhalation exposure. However, knowledge and awareness remain limited regarding the potential for isocyanate skin exposure to contribute to the development of isocyanate asthma. For example, the literature on occupational asthma rarely mentions isocyanate skin exposure as a potential risk factor or target for prevention (Nicholson et al. 2005; Tarlo and Liss 2005). Over the past several years there has been a growing, but largely unrecognized, collection of animal, industrial hygiene, clinical, and epidemiologic data related to isocyanate skin exposure and its role in the development of isocyanate sensitization and asthma. Our primary purpose in this article is to review and synthesize this multi-disciplinary literature to address several key unresolved issues, including the extent of isocyanate skin exposures in the workplace, the effectiveness of personal protective equipment, and most importantly, whether human skin exposure contributes to the development of isocyanate asthma. The findings may be relevant to the larger issue of the role of skin as an important underrecognized site of exposure and sensitization for other environmental allergens.

## Methods

### Definition of terms

The terms “skin exposure” and “dermal exposure” are used interchangeably to indicate exposure to the outermost layer of the epidermis or epicutaneous exposure, as is commonly done in the occupational and environmental literature. “Isocyanates” refers to diisocyanate monomers (two NCO groups) and their related polyisocyanates, which have similar health effects (Bello et al. 2004). The term “sensitization” can generate misunderstanding. “Sensitization” generally refers to priming of the immune system in response to a specific non-self antigen, a condition that involves immune memory, typically antigen-specific T cells and/or antibodies. Subsequent reexposure to the antigen can result in an immunopathologic adverse reaction, such as a Th2 (T-helper 2)-type acute allergic reaction or asthmatic response, or contact hypersensitivity-type reaction, such as allergic contact dermatitis (Sheaarer and Fleisher 2003). Others consider sensitization any immunologic memory of exposure, regardless of its pathogenic potential (i.e., a specific IgG response) (Abbas et al. 2000). Skin or respiratory sensitization typically refers to the route of exposure that results in systemic sensitization, rather than localized immune responses at those sites.

### Literature search

We have been active in the field of isocyanate research and have collected > 800 published and unpublished articles and documents from 1951 to the present; these articles and documents are related to isocyanates and span many disciplines. In addition, we performed computerized searches of the literature on Medline [1966 to the present (National Library of Medicine, Bethesda, MD)], National Institute for Occupational Safety and Health (NIOSH), Occupational Safety and Health Administration (OSHA) and U.S. Environmental Protection Agency (EPA) databases and Google (google.com) using the key words “isocyanate,” “diisocyanate,” “asthma,” “sensitization,” “exposure,” “dermal,” “skin,” “occupational,” “methylene diphenyl diisocyanate,” and other synonymous terms. Additional articles were identified from the reference lists of the selected relevant articles. We reviewed primarily English-language articles, as well as selected articles in German, Danish, and French. Human and animal articles that addressed isocyanate skin exposure, sensitization, and health effects were retained for further analysis. We also included clinical, epidemiologic, and biomarker studies and case reports that mentioned skin as a potential route of exposure or had low isocyanate air levels based on exposure data or work processes.

## Results and Discussion

### Health effects of isocyanate exposure

Isocyanates are considered potent respiratory allergens. Isocyanate asthma is the major health problem in isocyanate-exposed workers, affecting approximately 1–25% of the exposed population. The most important risk factor is isocyanate exposure, but the exposure characteristics and host factors involved remain unclear (Bernstein 1996; Wisnewski et al. 2006). Isocyanates can also cause hypersensitivity pneumonitis, contact dermatitis, and rhinitis, but these outcomes are less commonly reported (Baur et al. 1994; Musk et al. 1988).

Clinically isocyanate asthma presents similar to other types of allergic Th2-like asthma: Isocyanate asthma typically develops after repeated exposure for months to years, during which time sensitization to isocyanates is presumed to occur. Once sensitized, extremely low respiratory levels of isocyanate can elicit asthmatic responses. However, unlike for typical high molecular weight (MW) occupational allergens or environmental aeroallergens, research has failed to identify an isocyanate-specific immune response in most isocyanate asthmatics that indicates isocyanate Th2-like sensitization, such as a radioallergosorbent test (RAST) or skin prick test, thus hindering diagnosis. This difference may reflect involvement of non-IgE mechanisms in isocyanate asthma pathogenesis, and/or may be related to the NCO functional group common to all isocyanates that renders them ideal cross-linking agents (Wisnewski et al. 2006). Unlike high-MW allergens, isocyanates can react with amino (NH_2_, NH), hydroxyl, and sulfhydryl groups of various proteins and peptides, including albumin and keratin, to form a number of different hapten–protein complexes or antigens (Wisnewski et al. 1999, 2000). Human isocyanate skin-patch testing has been used to confirm sensitization in the uncommon person who develops contact hypersensitivity (allergic contact dermatitis) due to isocyanate skin exposure, but has not been helpful for detecting the Th2-like sensitization presumed to lead to isocyanate asthma (Kanerva et al. 2001). The mechanisms by which isocyanates cause asthma remain poorly defined, and lack of a good immunologic marker and unclear dose–response relationships have hindered diagnosis and prevention (Wisnewski et al. 2006). The primary focus here is on the potential for skin exposure to contribute to the development of isocyanate asthma rather than pathogenic mechanisms. However, a better understanding of the role of skin exposure may help address these key problems.

Several observations suggest that skin may also be an important site of exposure and sensitization. Isocyanate respiratory exposure alone, without any skin exposure, seems unlikely in most work settings. Isocyanate asthma occurs in settings with minimal documented respiratory exposures but clear potential for skin exposure, and splashes and spills have been reported by workers who subsequently develop isocyanate asthma (Bernstein et al. 1993; Lenaerts-Langanke 1992; Liss et al. 1988; Nemery and Lenaerts 1993; Zammit-Tabona et al. 1983).

### Workplace and environmental isocyanate exposures

**Figure 1:**
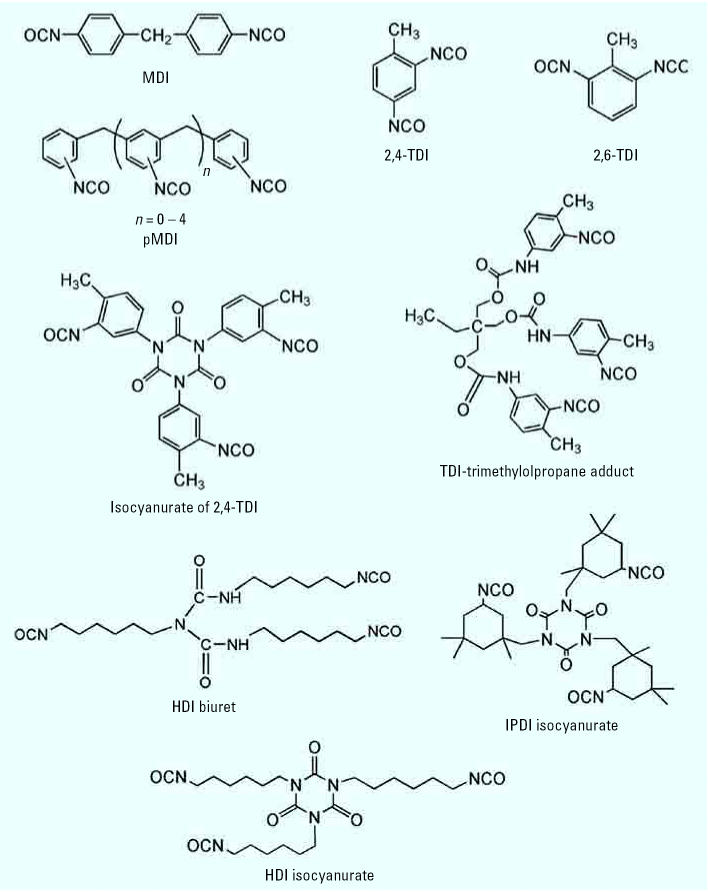
Chemical structures of selected isocyanates of commercial importance.

The chemical structures and important physicochemical properties of selected isocyanates are shown in Figure 1 and Table 1. The major commercial isocyanates are methylene diphenyl diisocyanate (MDI), toluene diisocyanate (TDI), and their nonvolatile polymeric forms pMDI and pTDI, followed by polymeric hexamethylene diisocyanate (pHDI) and isophorone diisocyanate (pIPDI). Isocyanates are reactive chemicals used extensively to make numerous polyurethane and other commercial products, such as poly-urethane foams, adhesives, and coatings. They are found in a wide range of industries, from construction to medical care. The increasing use of nonvolatile polyisocyanates has raised issues related to their measurement, exposure metrics, and regulation (Bello et al. 2004).

Occupational exposures to isocyanates occur primarily in the many end-use settings, as well as in primary production facilities where exposures are generally better controlled. The total number of workers currently exposed to isocyanates is not known. NIOSH’s estimate of 280,000 U.S. workers exposed or potentially exposed to isocyanates (NIOSH 1996) is undoubtedly higher today, given industry growth and new applications. Most commercial isocyanate products are complex isocyanate mixtures of variable molecular mass, volatility, and isocyanate content. Workplace exposures can occur in the form of isocyanate vapors, aerosols, or both, depending on the isocyanate type as well as the application method and other factors. Isocyanates are commonly mixed with various solvents, polyols, and other substances, such as catalysts and blowing agents, which may affect isocyanate reactivity, skin absorption, and health effects.

Although data are limited, environmental exposures to unreacted isocyanates are also possible. A number of consumer products contain unreacted isocyanates, such as glues, polyurethane coatings, and foam insulation; domestic use of such products on rare occasion has been reported to trigger asthma symptoms in individuals previously sensitized (Carroll et al. 1976; Dietemann-Molard et al. 1991; Peters and Murphy 1971). Based on patch testing, a few cases of allergic contact dermatitis have been reported with the use of consumer products made from isocyanates and polyurethanes (Alomar 1986; Morgan and Haworth 2003; Vilaplana et al. 1987). Certain biomedical products, such as standard orthopedic casting material, may be a potential source of skin exposure to unreacted isocyanates for patients and cast technicians (Legris et al. 1995). Polyurethane foams and packaging have been reported to contain very small amounts of unreacted isocyanates (Gagne et al. 2003; Krone et al. 2003), but it is unclear whether these products have any potential to result in skin exposure. Environmental isocyanate exposures can also potentially occur from the release of isocyanates into the environment from primary or secondary production facilities (Darcey et al. 2002; De Zotti et al. 2000; Orloff et al. 1998), during the transport or storage of isocyanates or polyurethanes (Allport et al. 2003), or during the thermal degradation of overheated polyurethane products (Boutin et al. 2006), but these exposures are rarely reported. Isocyanate releases from end-users, such as auto body shops located in or near residential neighborhoods, are also possible but rarely reported. It has been hypothesized that environmental skin exposure to polyurethanes in childhood has contributed to the increased prevalence of childhood asthma (Krone and Klingner 2005), but human isocyanate exposure from such products has not been documented, and there are numerous other likely causative factors.

The primary isocyanate exposure routes are through the respiratory tract and the skin. Historically, the focus has been on inhalation exposures. Increased use of less-volatile MDI and polymeric isocyanates, as well as improved hygiene practices, have resulted in reductions in inhalation exposures to volatile monomer (Bello et al. 2004), thus potentially increasing the relative importance of skin exposure. Isocyanate skin exposure could contribute a significant part of the total body burden. For example, 1% skin absorption of a small MDI droplet (10 mg) would deliver a dose approximately 4.5-fold (450%) higher than the inhalation exposure at the current short-term UK occupational exposure limit (15-min, 70 μg NCO/m^3^) or approximately 50% of the corresponding 8-hr (20 μg NCO/m^3^) standard, assuming 100% lung retention and a ventilation rate of 7 L/min (Bello et al. 2004).

### Measuring isocyanate skin exposure

Quantification of isocyanate skin exposure is important for research, prevention, and control. Assessment of skin exposure, in general, is much less developed than that of inhalation exposure. Skin exposure sampling methods typically are nonstandardized, have undergone limited validation, and can be technically challenging. Isocyanate skin sampling is further complicated by several factors, including the reactivity of NCO groups toward skin proteins, water, or other compounds, and the complexity of most isocyanate exposures. Biomarkers such as urinary metabolites, if available, could potentially be used to assess internal dose, but would not distinguish between skin and respiratory exposure.

Techniques that have been used to detect isocyanate skin exposure include SWYPE pads (Colormetric Laboratories Inc., Des Plains, IL) (Liu et al. 2000), wipes (Bello et al., in press), and tape stripping (Fent et al. 2006). These methods rely on removal of isocyanates from the skin—usually a period of time after initial exposure, and can underestimate exposure as a result of losses due to absorption, chemical reactions, and/or poor removal efficiency (Bello et al. 2005; Wester et al. 1999). Techniques that quantitate isocyanate deposited on the skin, such as reagent-impregnated patches, may overcome some of these limitations. Recently, extraction of isocyanates from contaminated gloves was used to measure hand skin exposure to isocyanates (Pronk et al. 2006). Application of these skin exposure methods in the workplace has been limited, and findings have not been compared or validated. Workplace isocyanate skin exposure assessment is further complicated by the frequently sporadic nature of such exposures.

### Workplace isocyanate skin exposure

Numerous isocyanate end uses, such as spraying and application of foams and adhesives, provide opportunity for isocyanate skin exposure from deposition of aerosols and/or absorption of vapors. Typical workplace isocyanate exposure levels are not irritating and give few warning signs, and skin protective equipment may not be worn, even when respiratory protection is used (NIOSH 1999). Skin exposure may result from direct contact of unprotected skin or the failure of personal protective equipment, such as gloves. Opportunities for isocyanate skin exposure, such as spills, cleanup, and contact with contaminated equipment, are well known to workers and field researchers. For example, NIOSH Health Hazard Evaluations of a variety of work settings have described workers with skin contact to isocyanates or uncured polyurethane products (NIOSH 1998, 1999).

Isocyanate products that are not fully cured are another potential source of isocyanate skin exposure (Bello et al., in press). It is commonly believed that isocyanate-containing products polymerize rapidly, and once the product appears hardened, no unbound isocyanate species remain on the surface. However, there are few published data confirming this. A recent study demonstrated that curing proceeded more slowly than expected, with unbound isocyanate species detected on painted surfaces for prolonged periods of time (days to weeks) (Bello et al., in press). Thus, handling recently cured isocyanate products could be a source of isocyanate skin exposure. In addition, release of free isocyanates has been reported after heating of cured isocyanate products, as can occur when grinding, cutting, or sanding such products (Alliance for the Polyurethanes Industry 2005; Boutin et al. 2006; Littorin et al. 2002). Such tasks could represent another possible source of isocyanate skin exposure.

In spite of observational documentation of workplace isocyanate skin exposures, published data documenting such exposure are surprisingly limited. Isocyanate skin exposure has been documented qualitatively with colorimetric techniques in several work settings (Liu et al. 2000; NIOSH 1998). Recent studies have begun to quantitate such exposures. Fent et al. (2006) detected HDI monomer on tape strips from an auto-body painter’s unprotected skin. Pronk et al. (2006) recently reported isocyanate exposure under gloves on both hands of auto body and industrial spray painters, with the highest median hand exposure detected during paint mixing (207 and 63 μg NCO, respectively).

### Effectiveness of personal protective equipment

Gloves and protective clothing remain a primary means of preventing skin exposure in the workplace, in addition to engineering and work practice controls. Gloves and protective clothing are presumed to protect against isocyanate skin exposure, with nitrile gloves considered preferable to latex. However, data on the workplace performance of protective gloves and clothing are limited, and there is evidence that isocyanates (Liu et al. 2000; Pronk et al. 2006) and solvents (Collin-Hansen et al. 2006) can be detected underneath gloves.

### Skin absorption of isocyanates

Animal studies employing radiolabeled ^14^C-MDI have demonstrated absorption of MDI after skin exposure (Leibold et al. 1999; Vock and Lutz 1997). However, quantitative data are limited and may have underestimated absorption due to technical issues, such as isocyanate binding to the dressing and skin. Studies documenting the disappearance of isocyanates from guinea pig skin with infrared spectroscopy also support isocyanate skin absorption (Bello et al. 2006).

We are not aware of published data directly confirming isocyanate skin absorption in humans; however, a number of studies indirectly demonstrate isocyanate skin absorption. For example, HDI-conjugated keratins have been identified from human skin biopsies obtained after epicutaneous application of HDI (Wisnewski et al. 2000). Patch testing with isocyanate (0.1–1%) can elicit an isocyanate-specific skin contact hypersensitivity reaction that implies isocyanate skin absorption.

Studies investigating urinary biomarkers of isocyanate exposure (the corresponding diamines) have provided additional indirect evidence for isocyanate skin uptake (Creely et al. 2006; Kääriä et al. 2001; Maitre et al. 1996). Elevated levels of these urinary biomarkers have been detected in workers, in spite of very low or nondetectable documented inhalation exposures. Greater than 2-fold higher urinary metabolite concentrations have been reported for operators with likely skin contamination compared to those without (Creely et al. 2006). Maitre et al. (1996) measured urinary hexamethylene diamine (HDA) of two HDI-exposed coworkers. Both workers had similar inhalation exposure as confirmed by air measurements, but one had considerably greater HDI skin contact and a 10-fold increase in urinary levels of HDA. The authors concluded that HDI seemed to be readily absorbed through the skin.

Human isocyanate skin absorption likely depends on a number of factors, in addition to the extent of isocyanate skin exposure, and may vary between isocyanates because of differences in their physical and chemical properties, including molecular mass, fat solubility, and chemical reactivity. Skin absorption can be enhanced if the barrier properties of skin have been damaged, such as can occur with eczema, cuts, hand washing, cosmetics applications (shaving, waxing), and other conditions (Moody and Maibach 2006; Smith Pease et al. 2002). In addition, coexposures such as solvents used in the production of polyurethane foams, coatings, and spray applications can be absorbed through the skin (Boman and Maibach 2000). Such solvents may enhance isocyanate absorption and also break through the gloves.

### Skin sensitization

Numerous substances—primarily low-MW haptens, such as metals and chemicals, and less commonly proteins—are known to initiate immune responses in the skin, most commonly hapten-induced contact hypersensitivity (Kimber and Dearman 2002). Contact hypersensitivity (allergic contact dermatitis) following skin exposure to isocyanates is well documented in animals and in the clinical dermatologic literature, with sensitization confirmed with patch testing (Goossens et al. 2002; Herrick 2002). Allergic contact dermatitis has been reported following skin exposure to isocyanates and polyurethane products in a number of different workplace and non-occupational settings, but has not been considered common, and is rarely reported in workers with isocyanate asthma (Alomar 1986; Frick et al. 2003; Goossens et al. 2002; Wilkinson et al. 1991). However, allergic contact dermatitis may be more common than suspected because symptoms can be mild, workers being evaluated for asthma are frequently not asked about skin problems, and patch testing can be falsely negative (Frick et al. 2004; Goossens et al. 2002).

Much less is known about the role of skin exposure in inducing Th2-type immune responses seen in asthma. Recent animal studies have documented that skin exposure to proteins such as ovalbumin or peanuts can induce systemic Th2-type sensitization and subsequent asthmatic responses (Herrick et al. 2003; Strid et al. 2005). Limited clinical and epidemiologic studies also support a role for skin exposure to allergens in the development of Th2-type sensitization and asthma, or of other immunologic lung diseases such as chronic beryllium disease (Cummings et al. 2006; Lack et al. 2003; Saloga and Knop 2000; Smith Pease et al. 2002; Tinkle et al. 2003).

### Isocyanate skin exposure, sensitization, and asthma

#### Animal models

Animal models using the three major isocyanates MDI, TDI, and HDI have all employed skin exposure to induce sensitization, with subsequent inhalation challenge, to create an asthmatic response in the lungs (Table 2). Skin sensitization with other chemicals, such as trimellitic anhydride, followed by inhalational challenge has also produced asthmatic responses (Vanoirbeek et al. 2006; Zhang et al. 2004).

Different sensitization and challenge protocols (variable doses, frequency, timing, formulation, and route of exposure) using different isocyanates have resulted in variable pulmonary responses. In spite of these differences, several common themes emerge from these animal studies. First, skin has been a very effective route of inducing sensitization, sometimes more effective than inhalation (Ban et al. 2006; Rattray et al. 1994). For example, Ban et al. (2006) recently evaluated several different TDI sensitization scenarios (inhalation, subcutaneous, topical) and challenge protocols (vapor, tracheal instillation) in mice. Topical sensitization followed by tracheal instillation of TDI most closely reproduced the Th2-type lung inflammatory response seen in human asthma, whereas sensitization with vapor TDI was not effective.

Of note, these isocyanate animal models also demonstrate that a single one-time or two-time skin exposure with relatively low concentrations of isocyanates can be sufficient to induce sensitization (Herrick et al. 2002; Karol et al. 1981; Rattray et al. 1994). Additionally, several models have shown paradoxical dose–response relationships, such that a lower skin sensitization dose can result in greater lung inflammation upon inhalation challenge than a higher sensitization dose, and that isocyanate-specific antibody responses may not correlate with asthmatic responses (Herrick et al. 2002; Vanoirbeek et al. 2004). The effective skin sensitizing doses in these studies, typically in the order of 1–100 μmol NCO (Table 2) delivered as diluted 1–10% isocyanate solution, represent a few droplets of a diluted isocyanate product. Comparable exposures likely occur in the workplace.

Thus, several different animal models in more than one species clearly demonstrate that isocyanate skin exposure can induce systemic sensitization, which with subsequent inhalation exposure can lead to asthma. Control experiments in which animals received only skin exposure demonstrate that isocyanate skin exposure alone does not cause asthmatic responses (Herrick et al. 2002; Karol et al. 1981; Vanoirbeek et al. 2004). Although issues of comparability with humans are inevitable, such as differences in skin permeability, much can be learned from animal models regarding exposure–response relationships and the mechanisms that lead to skin sensitization and asthma.

#### Human studies

Although patch testing has confirmed contact hypersensitivity following human isocyanate skin exposure, direct evidence that skin exposure leads to Th2-type sensitization and the subsequent development of asthma is limited. As noted above, there is no good test to identify isocyanate sensitization in humans. Isocyanate-specific IgE is present in less than half of isocyanate asthmatics (Mapp et al. 2005; Wisnewski et al. 2006), and isocyanate-specific IgG appears to be indicative primarily of exposure (Liss et al. 1988; Welinder et al. 1988; Wisnewski et al. 2006). Isocyanates are considered a potent respiratory sensitizer in humans, based largely on circumstantial evidence rather than on clear demonstration that respiratory exposure alone (without associated skin exposure) leads to isocyanate asthma.

Indirect evidence from a growing number of case reports and clinical and epidemiologic studies suggests that isocyanate skin exposure occurs in the workplace and can increase the risk for sensitization and isocyanate asthma. Isocyanate asthma and/or sensitization (MDI-IgE) have been reported in several case studies of workers who applied MDI-based orthopedic casts (Donnelly et al. 2004; Legris et al. 1995; Sommer et al. 2000). Allergic contact urticaria and asthma following direct hand contact with MDI glue has been documented based on a positive MDI-IgE, MDI patch test, and MDI inhalation challenge (Valks et al. 2003). Consistent across these case reports is the development of MDI asthma in settings where skin exposure to MDI occurred and where MDI air levels, if measured, were non-detectable or extremely low and opportunity for MDI respiratory exposure was very limited. These cases strongly suggest that skin exposure was the predominant exposure route and contributed to the development of isocyanate sensitization and asthma. They also demonstrate that isocyanate asthma can occur in settings where measured isocyanate respiratory exposures are below the level of detection, even when sensitive analytical methods are used.

Skin exposure has also been implicated as a risk factor for isocyanate asthma in several epidemiologic studies of MDI-exposed workers, in which measured inhalation exposures were all very low or nondetectable. Petsonk et al. (2000) investigated a group of new workers exposed to MDI resins in a facility designed for minimal airborne exposures and noted new asthma-like respiratory symptoms in 27% of the workers with the highest potential for MDI exposure. These symptoms were associated with liquid MDI skin exposure, as evaluated by worker questionnaires and workplace observations.

Leanerts-Langanke (1992) described an investigation of about 500 coal mine workers who used MDI for rock consolidation. Quantitative air sampling results were at or below the detection limit (1 ppb). MDI skin exposure was reported in about half the workers; MDI-IgE was detected in several workers; and 2 were diagnosed with isocyanate asthma based on a positive specific challenge. Elevated levels of MDI metabolites in urine were found in 6 of 8 workers seen after “massive skin contamination.” The authors concluded that skin could be an important route of exposure, leading to sensitization and asthma.

Bernstein et al. (1993) studied a cohort of 243 workers in a urethane molding plant that consistently maintained low MDI airborne exposures (< 5 ppb). Isocyanate asthma and/or sensitization (MDI-IgE) was diagnosed in several workers, most of whom were reported likely to have had MDI skin exposure.

Our group has characterized workplace isocyanate exposures in a population of > 200 auto body shop workers with isocyanate-specific immune responses but without documented isocyanate asthma (Redlich et al. 2001; Woskie et al. 2004). Respiratory and skin exposures were estimated for each of the workers, based on exposure algorithms. HDI-specific IgG, present in 21% of the workers, was strongly associated with inhalation exposure, but skin exposure also contributed (Stowe et al. 2006). We have also detected MDI-specific IgG in > 30% of about 100 workers in a factory that uses MDI to produce polyurethane coated fabrics. MDI air monitoring data have consistently been very low, and MDI skin exposure has been documented by worker questionnaires and direct observation (Liss et al. 2006). In both work settings, isocyanate skin exposure appeared to contribute to the development of isocyanate-specific IgG, which has been associated with isocyanate exposure (Welinder et al. 1988; Wisnewski et al. 2004; Ye et al. 2006).

Studies investigating the respiratory exposure conditions that lead to isocyanate asthma also suggest a potential role for skin exposure. Fewer cases of isocyanate asthma have generally been reported in settings with lower respiratory exposures, but cases continue to occur in settings with consistently low reported air levels (Baur 2003; Tarlo et al. 1997). Skin exposure (Bernstein et al. 1993; Leanerts-Langanke 1992) or intermittent peak exposures, which could also entail both respiratory and skin exposure (Tarlo et al. 1997), have been considered important contributing factors in such cases. Typically, such exposures are unpredictable and frequently accidental, making them difficult to investigate and quantify.

For cutaneous allergen exposure in general, the likelihood of sensitization in a susceptible person is thought to depend on several factors including the total dose and concentration of allergen, skin surface area, and the frequency of repeated contact with the skin, with determinants of individual susceptibility remaining poorly defined (Basketter et al. 2006; Boukhman and Maibach 2001). There are insufficient data to address isocyanate skin exposure–response relationships in humans; however, as noted above, in animal models relatively small skin doses can induce sensitization, and dose–response relationships may be variable.

### Regulatory standards for isocyanate skin exposure

The regulatory framework for skin exposure to chemicals is underdeveloped compared with inhalation exposures. Chemicals that pose a health hazard through skin exposure are commonly assigned two qualitative descriptors: a “skin” notation, referring to absorption of the chemical through the skin, and/or a “sensitizer” notation for an agent with the potential to produce sensitization regardless of the exposure route (respiratory, skin, or conjunctiva) [American Conference of Governmental Industrial Hygienists (ACGIH) 2006].

In the United States, although NIOSH recommends prevention of isocyanate skin exposure, skin exposure is not regulated. No “skin” notation exists for diisocyanates or polyisocyanates, except for the NIOSH (2005) recommendation for IPDI.

Although data confirming the risks of human isocyanate skin exposure remain limited, there is sufficient information to recommend prevention of skin exposure. Such recommendations are now being made in material safety data sheets and are beginning to appear in the medical literature (Bakke et al. 2006; Baur 2003). Wider dissemination and improved hazard communication of this information by occupational and environmental health professionals, as well as better personal protection among workers, are needed. Inclusion of “skin” notation may encourage such protection for all isocyanates (diisocyanates and polyisocyanates).

### Research needs

In this review we highlight several important areas for further research regarding isocyanate skin exposure, ideally using multidisciplinary approaches involving animal models and clinical and epidemiologic investigations. Such approaches should lead to a better understanding of the mechanistic pathways that result in isocyanate asthma and the role of skin exposure in this process. A key research need not unique to issues regarding skin exposure is the development of a good marker of isocyanate sensitization or “pre-clinical” asthma that correlates well with the subsequent development of asthma. Such a marker would greatly enhance isocyanate research, including elucidation of exposure–response relationships, and facilitate diagnosis and prevention.

There is a need to better assess isocyanate skin exposures in the workplace and other environments and to incorporate these exposure data into epidemiologic and clinical studies. The typically more sporadic nature of such exposures further complicates real-world exposure assessment and requires algorithms that employ a combination of daily activities (diaries), questionnaires, and task-based exposure data. Development of route-specific bio-markers, such as those specific for skin or lung, would greatly facilitate isocyanate exposure assessment. Skin exposure methodologies and biomarkers of exposure can be further developed and validated using integrated animal models.

Numerous host and environmental determinants of isocyanate skin exposure have barely been investigated. Isocyanate skin absorption likely depends on various factors including molecular size and coexposures (e.g., polyols, solvents, and other additives), which could enhance absorption. The role of host factors, such as history of eczema and hand washing, warrant further investigation, as does the effectiveness of gloves, protective clothing, and other preventive strategies.

Given the widespread and growing use of polyurethane consumer products, research is also needed to investigate potential environmental exposures from these consumer products, as well as exposures that may occur when isocyanate products are manipulated (ground, cut, drilled) or undergo thermal degradation as in fires. Further research investigating the curing process is also warranted because incompletely cured products could be a potential and unexpected source of skin exposure (Bello et al., in press).

## Conclusion

Isocyanates, primarily diisocyanate monomers and polyisocyanates, are a leading cause of occupational asthma. Human skin exposure to isocyanates has been underrecognized and can occur in various workplace and environmental settings. Multiple lines of evidence from animal studies and clinical, epidemiologic, and biomarker studies, as well as anecdotal evidence, indicate that in certain exposure settings human skin likely is an important route of isocyanate exposure and can contribute to the development of isocyanate asthma. This presumably occurs by isocyanate skin exposure inducing systemic sensitization, which then leads to isocyanate asthma after inhalation exposure; however, the mechanistic pathways involved remain poorly defined. Further research is needed to address issues regarding isocyanate skin exposure in the growing polyurethane industry. In spite of substantial research needs, sufficient evidence already exists to justify greater emphasis on the potential risks of isocyanate skin exposure and the need to prevent such exposures. We conclude with the visionary statement of Munn (1965):

The more one knows about these fascinating compounds [isocyanates] the more fascinated one becomes. So diverse are their uses, it is obvious that they are here to stay, and that their use will increase. So numerous have been the accounts of their effects, it is obvious not merely they are hazardous but that the nature and extent of their hazard has not always been fully appreciated.

## Figures and Tables

**Table 1:** Selected physicochemical properties of commercially important isocyanates.

Isocyanate^a^	MW (g/mol)	VP (mm Hg)^b^	Volatility^c^	1 ppm = *x* mg/m^3^
HDI	168.2	0.02	Very high	6.88
TDI	174.2	0.02	Very high	7.13
IPDI	222.3	0.0003	Moderate	9.09
MDI	250.3	0.000005	Very low	10.24

VP, vapor pressure.

a All isocyanates are liquids at room temperature, except for MDI.

b VP at 20°C, except for MDI (25°C); data from Streicher et al. (1994).

c The higher the volatility, the shorter the residence time of isocyanates on the skin; most polymeric isocyanates (MW ~ 500) are nonvolatile at room temperature.

**Table 2:** Selected animal models of isocyanate asthma: sensitization via skin.

		Sensitization via skin		Inhalation challenge		
Compound, reference	Animal	Frequency/form	Dose (μmol NCO)	Frequency/form/route/timing	Dose	End points (inflammation, airway responsiveness, isocyanate antibodies, other)
TDI						
Karol et al. 1981	Guinea pig	Single; 1/day × 50 μL 1, 10, 25, 100% in OO	Various; ~ 7, 70, 174, 1,390	Once/inhalation/TDI vapor and TDI-GSA conjugate aerosol, 2 weeks later	5 ppb TDI vapor and 12 μg/L air TDI-GSA	↑ Respiratory rate, ↑ TDI-IgE antibodies
Erjefält and Persson 1992	Guinea pig	Twice; days 1 and 8; 1/day × 2 days × 100 μL 20% TDI in EA/OO	2 × ~ 278 = 556	Once; tracheal, TDI, day 15	~ 1.4–4 pmol NCO	↑ Plasma exudation in lungs (^131^I-albumin)
Scheerens et al. 1999	Mice BALB/c	Short exp: 2/day × 2 days; long exp: 1/week × 6 weeks; both: × 200 μL 1% TDI in Ace/OO	Short exp: 2 × 55.6 = 111 Long exp: 6 × ~ 28 = 167	Once; intranasal, 20 μL 1% TDI in EA/OO and ear 20 μL 0.5% in Ace; day 8 or day 42	Intranasal 2.8 μmol NCO + ear 1.4 μmol NCO	Short: ↑ TDI-IgG, long: ↑ TDI-IgE and TDI-IgG, ↑ *in vivo* tracheal hyperresponsiveness
Vanoirbeek et al. 2004	Mice BALB/c	Variable; once (1% TDI) or 1/day × 3 days (1, 2, 3); 40 μL 0.3% or 3% TDI in Ace/OO, + repeat on day 7	1% TDI: 5.6 × (1+1) = 11.2; 0.3% TDI: ~ 1.7 × (3 + 1) = 6.7; 3% TDI: 16.7 × (3 + 1) = 66.8	Once; intranasal, 20 μL 0.1% TDI in Ace/OO, day 10	0.28 μmol NCO	↑ Airway responsiveness, ↑ mixed Th1/Th2 lung inflammation, ↑ total IgE, greater response with lower skin dose; timing and freq skin exposure important
Ban et al. 2006	Mice BALB/c	Single; 50 μL 1% TDI in Ace/OO on day 0, then tracheal instillation with 50 μL 0.2% TDI in OO on day 5	7 on skin + 1.4 tracheal instillation	Trachea instillation with 50 μL 0.1% TDI in OO, on days 18, 28, 31	3 × 0.7 = 2.1 μmol NCO	Lung Th2 inflammation, ↑ TDI-IgE, only skin sensit induced Th2 asthma-like responses
HDI						
Herrick et al. 2002	Mice BALB/c	Twice; 50 μL 0.01 or 1% HDI in 4:1 A:OO on days 0 and 7	1.2 and 12.4	Intranasal; 50 μL HDI-MSA (2 μg/μL) on days 14, 15, 18, 19	100 μg	Lung and BAL Th2 inflammation, ↑ IgE, ↑ HDI-IgG, lower sensit dose, more lung inflammation
MDI						
Rattray et al. 1994	Guinea pig	Single, variable; 400 μL of 10, 30, or 100% MDI in CO	320, 959, 3,195	Once; inhalation of MDI aerosol on day 21	26–36 mg/m^3^	↑ Respiratory rate, ↑ MDI-IgG1, inhalation failed to induce sensit

Abbreviations: ↑, increase; Ace, acetone; Alb, albumin; BAL, bronchoalveolar lavage; CO, corn oil; EA, ethyl acetate; exp, exposure; Freq, frequency; GSA, guinea pig serum albumin; MSA, mouse serum albumin; OO, olive oil; sensit, sensitization.
